# Lessons learned from revision procedures: a case series pleading for reinforcement of the anterior hiatus in recurrent hiatal hernia

**DOI:** 10.1007/s00464-024-10703-3

**Published:** 2024-04-02

**Authors:** Jorrit H. Geerts, Job W. A. de Haas, Vincent B. Nieuwenhuijs

**Affiliations:** Department of Surgery, Isala, Zwolle, The Netherlands

**Keywords:** Hiatal hernia, Recurrence location, Laparoscopy, Mesh reinforcement, Case series

## Abstract

**Background:**

Hiatal Hernia (HH) is a common structural defect of the diaphragm. Laparoscopic repair with suturing of the hiatal pillars followed by fundoplication has become standard practice. In an attempt to lower HH recurrence rates, mesh reinforcement, commonly located at the posterior site of the esophageal hiatus, has been used. However, effectiveness of posterior mesh augmentation is still up to debate. There is a lack of understanding of the mechanism of recurrence requiring further investigation. We investigated the anatomic location of HH recurrences in an attempt to assess why HH recurrence rates remain high despite various attempts with mesh reinforcement.

**Methods:**

A retrospective case series of prospectively collected data from patients with hiatal hernia repair between 2012 and 2020 was performed. In total, 54 patients with a recurrent hiatal hernia operation were included in the study. Video clips from the revision procedure were analyzed by a surgical registrar and senior surgeon to assess the anatomic location of recurrent HH. For the assessment, the esophageal hiatus was divided into four equal quadrants. Additionally, patient demographics, hiatal hernia characteristics, and operation details were collected and analyzed.

**Results:**

54 patients were included. The median time between primary repair and revision procedure was 25 months (IQR 13–95, range 0–250). The left-anterior quadrant was involved in 43 patients (80%), the right-anterior quadrant in 21 patients (39%), the left-posterior quadrant in 21 patients (39%), and the right-posterior quadrant in 10 patients (19%).

**Conclusion:**

In this study, hiatal hernia recurrences occured most commonly at the left-anterior quadrant of the hiatus, however, posterior recurrences were not uncommon. Based on our results, we hypothesize that both posterior and anterior hiatal reinforcement might be a suitable solution to lower the recurrence rate of hiatal hernia. A randomized controlled trial using a circular, bio-absorbable mesh has been initiated to test our hypothesis.

Hiatal hernia (HH) is a common structural defect of the esophageal hiatus with a variety of symptoms ranging from heartburn to dysphagia. Most of these hernias are small, with approximately 95% consisting of the sliding type (type 1 hernia) and often being asymptomatic [1, 2]. Treatment consists of lifestyle changes and medical treatment, with surgery being utilized in cases where the aforementioned proves ineffective. Although laparoscopic hernia repair with crural suturing and fundoplication has become standard practice, recurrent HH after surgical intervention is common, with reported recurrence rates ranging from 10 to 50% [3, 4]. These high recurrence rates have led to the usage of mesh reinforcement additionally to cruroplasty in an attempt to reduce recurrences. It is suggested that prosthetic mesh augmentation might reduce recurrence rates [5–7]. In the PRIME-trial, conventional posterior cruroplasty, supplemented by anterior cruroplasty if deemed necessary, was compared to posterior cruroplasty followed by posterior, non-absorbable mesh (TiMesh®) augmentation. Short-term results, however, did not show a reduced recurrence rate in the mesh-group [8]. Additionally, a similar study by Watson et al. failed to show superiority in the posterior, prosthetic mesh group at 5-year follow-up [9].

The wide range of reported results raises the question whether mesh reinforcement is helpful. First, it is vital to understand the basic anatomy and pathology. The esophageal hiatus is formed by two muscle fiber bundles called the left and right pillars, both originating from the right crus of the diaphragm. The right crus reaches from the retroperitoneum posteriorly to the central tendon of the diaphragm anteriorly [10, 11]. The phrenoesophageal membrane helps to stabilize the gastroesohageal junction (GEJ) in the abdominal cavity, inferior to the hiatus. Naturally, the pressure in the abdominal cavity is around 5 to 14 mmHg, while the pressure in the thoracic cavity is much lower at − 4 to − 16 mmHg, resulting in a pressure difference of 9 to 30 mmHg [12, 13]. This pressure gradient is transmitted through the gap between the esophagus and hiatus, resulting in stress on the surrounding tissues. Consequentially, an HH develops when the stress to the hiatus and phrenoesophageal ligament exceeds their tensile strength [13]. From here, it becomes apparent how factors like obesity, past childbearing and collagen disorders increase the risk of developing an HH by further increasing the intra-abdominal pressure.

Since the aforementioned PRIME-trial, amongst others, failed to show superior surgical outcomes in the mesh group, we questioned the reasons for the failure of these posteriorly targeted meshes [8]. We identified a lack of understanding on the development of recurrent HH. A better view on the timing and location of HH recurrences is needed to explain the failure of the recent reinforcement attempts. Since mesh reinforcement often proves ineffective, perhaps we are not placing our reinforcement at the optimal location. This study was conducted after previous reports raised questions about the precise anatomic location of the recurrent HH, [14, 15]. In this study, we aimed to analyze the anatomic location of hiatal hernia recurrences after primary HH repair at the level of the esophageal hiatus.

## Methods

A retrospective, single-center, consecutive case series was conducted in Isala Zwolle, a large tertiary referral center for HH repair in the Netherlands with a caseload of approximately 120 procedures per year. The local ethics committee approved the study.

Data for this study were collected prospectively from 2012 until 2020 and stored in an electronic database (Research Manager). All patient who underwent revisional surgery for recurrent hiatal hernias were selected from this database and analyzed for this study. Two dedicated gastrointestinal surgeons with extensive experience in HH repair perform all primary and revision procedures in our hospital. Operations were recorded on video at every procedure and electronically stored in patient files (RVC Clinical Assistant) as per standard clinical practice. Revisional surgery cases conducted for recurrent gastro-esophageal reflux disease (GERD) without a significant hiatal hernia were excluded.

### Primary operation

Patient characteristics, etiology and data from the primary operation were analyzed in detail. These data include age, gender, BMI, operation indication (GERD or HH), type of primary HH, open or laparoscopic approach, type of crural repair (posteriorly only or both posteriorly and anteriorly) and number of sutures used, usage and location of mesh reinforcement and type of fundoplication (180 °C anterior, Toupet or Nissen). We acknowledge that the terms crural repair and cruroplasty are technically incorrect since the esophageal hiatus is formed by the left and right pillars of the right crus. The suturing of the pillars is therefore not a real crural repair. However, for the purpose of comparison to the existing literature we decided to use the common terminology cruroplasty and crural repair which refer to suturing of both pillars of the right crus.

### Classification of recurrence and location of recurrence

All operation notes, preoperative imaging and intraoperative video clips of the laparoscopic revisional procedures were reviewed to determine the type and location of the recurrence. To score the type of recurrence, the classification described by Duranceau was used [16]. In this classification, a type I HH (sliding type) consists of only upwards displacement of the GEJ above the level of the diaphragm. A type II HH consists of herniation of the gastric fundus into the mediastinum while the GEJ remains at its normal location. The type III HH involves both the GEJ and gastric fundus herniating through the esophageal hiatus, and a type IV HH involves herniation of other abdominal viscera into the mediastinum. To score the location, the hiatus and the diaphragm crurae were divided in four equal quadrants (left-anterior, right-anterior, left-posterior and right posterior) (Fig. 2). The quadrants were scored on involvement in recurrent hiatal herniation. Findings from video sequence analyzation was leading in cases were type or location of recurrence were inconsistent. If video sequences were not available, data from operation notes were leading if inconsistent with preoperative imaging. Video analysis was performed separately by both a surgical registrar and the senior surgeon, who performed all revision operations. In case of disagreement on the assessment of the HH recurrence location or type, the video has been reviewed together once more to come to a consensus.

### Statistics

Demographics and data variables were summarized with descriptive statistics. Normal distributed data were presented as mean with standard deviation (SD), while non-parametrical data were presented as medians with interquartile ranges (IQRs).

This case series has been reported in line with the PROCESS Guideline [17].

## Results

### Demographic characteristics

Demographic and preoperative data are presented in Table 1. Fifty-four patients underwent repair of a recurrent HH in the study period. Thirty-four of these patients (63%) were female, twenty were male (37%). Mean age ± SD was 63 ± 9.8 (range 34–77). Mean BMI ± SD was 27 ± 4.2 (range 22–36).

**Table 1:** Patient demographics and data on primary HH repair

Characteristics		Number of patients data available, *n* (%)
Age, y, mean ± SD	63 ± 9,8	54 (100)
Female, *n* (%)	34 (63)	54 (100)
BMI, kg/m2, mean ± SD	27 ± 4,2	54 (100)
Primary operation indication		54 (100)
Gastro-esophageal reflux disease	16 (30)	
Hiatal hernia	38 (70)	
Primary hiatal hernia type, *n* (%)		47 (87)
No hiatal hernia	2 (4)	
Type I	5 (11)	
Type II	2 (4)	
Type III	15 (32)	
Type IV	23 (49)	
Operation type, *n* (%)		54 (100)
Laparoscopic	48 (89)	
Open	5 (9)	
Converted	1 (2)	
Crural repair, *n* (%)		45 (83)
Posterior	24 (53)	
Anterior and posterior	21 (47)	
Posterior sutures, median [range]	3 [2–6]	
Anterior sutures, median [range]	1 [1–5]	
Fundoplication, *n* (%)		51 (94)
None	2 (4)	
180 deg anterior	36 (71)	
270 deg posterior (Toupet)	3 (6)	
360 deg circular (Nissen)	9 (17)	
Collis-Nissen	1 (2)	
Mesh reinforcement of hiatal repair, *n* (%)		54 (100)
No mesh used	51 (94)	
Posterior	3 (6)	
Anterior	1 (2)	

### Primary operation

Data from the primary operation are listed in Table 1. The primary operation indication was symptomatic HH in the majority of cases (38; 70%), whereas in 16 (38%) patients it was recurrent GERD. The primary HH classification was available in 47 patients (87%). Five patients (11%) had a type I hiatal hernia, two patients a type II (4%), 15 patients (32%) had a type III and 23 patients (49%) a type IV hiatal hernia. There was no hiatal hernia found at the primary procedure in two patients (4%). Both the primary and revision operation were conducted in our center by the same surgical team in 38 cases (70%), while the primary operation was conducted in another hospital in 16 cases (30%). These patients were referred to our center for a revisional operation. The primary operation was completed laparoscopically in 48 patients (89%), primary open in five patients (9%) and in one (2%) patient initial laparoscopic procedure was converted to open. The operation records of the primary procedures were available in 48 patients (89%). The technique of the crural repair was in all cases a primary suture repair posterior to the esophagus with non-absorbable sutures; in 21 cases (47%), an additional crural repair anterior of the esophagus was conducted. In three patients (6%), the hiatal repair was reinforced with a non-absorbable mesh posterior (2 patients, 4%) or anterior (1 patient, 2%) to the esophagus. Fundoplication was performed in 49 patients (89%), 37 of those (76%) were 180° anterior fundoplications, 9 (17%) were Nissen fundoplications, 3 (6%) were Toupet fundoplications and one (2%) was a Collis-Nissen fundoplication.

### Type and location of recurrent hiatal hernia

**Fig. 1:**
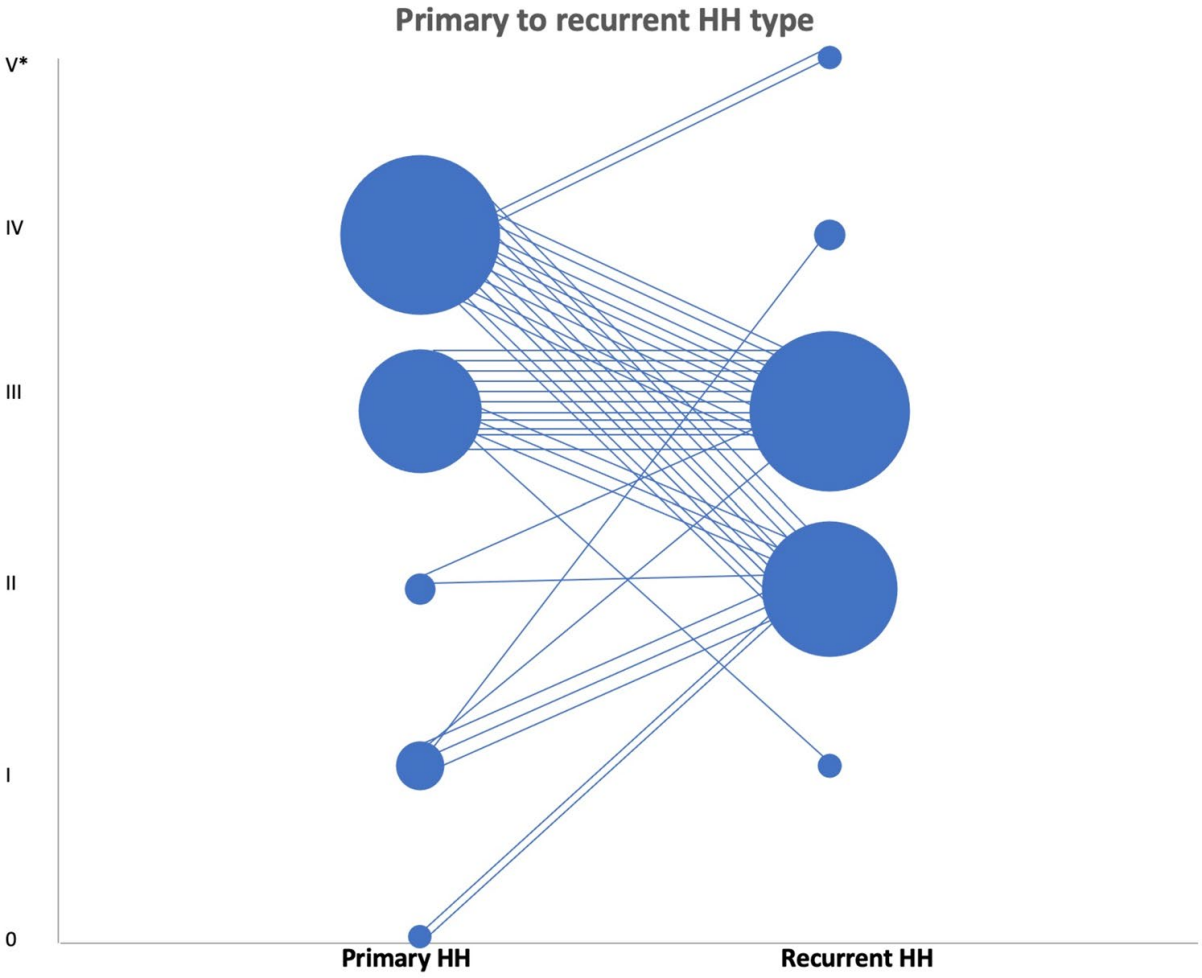
Graphic display of the association between primary and recurrent HH types. V* refers to crural disruption

Intraoperative findings from the revisional procedures are presented in Table 2. The median length of time between the primary operation and the revisional procedure was 25 months (IQR 13–95, range 0–250). Operation notes and preoperative imaging were available in all procedures. Intraoperative video clips were available in 48 cases (89%). One patient (2%) had a type I recurrence, 19 patients (35%) had a type II recurrence, 28 patients (52%) a type III recurrence and 4 patients (7%) a type IV recurrence. In two patients (4%) with an early recurrence (within a week from the primary operation), the type of recurrence remained unclassified as complete disruption of the crural repair was noted. Table 3 further classifies the association between the reported primary and recurrent HH types. These findings are graphically shown in Fig. 1 where each primary HH type is linked to the reported recurrent HH type. It should be noted that in Fig. 1, the cases of crural disruption are classified as V*. Cases in which the primary HH type was unknown were left out of Fig. 1.

**Table 2:** Intraoperative findings from revision procedure noted from intraoperative video clips

Analysis of revision procedures	
Video sequence available	48 (89)
Preoperative imaging available	54 (100)
Operation notes available	54 (100)
Time primary operation—revision surgery (months), median [range]	25 [0–250]
Type of recurrent HH, *n* (%)	
I	1 (2)
II	19 (35)
III	28 (52)
IV	4 (7)
Disrupted cruroplasty	2 (4)
Quadrant involved in recurrent hiatal hernia, *n* (%)	
Right anterior	21 (39)
Left anterior	43 (80)
Left posterior	21 (39)
Right posterior	10 (19)
Involvement of more than one quadrant, *n* (%)	37 (69)

**Table 3:** Recurrent HH types classified by primary HH type

Recurrence type per primary HH		54 (100)
Primary type I		5 (9)
Recurrence type I	0 (0)	
Recurrence type II	3 (60)	
Recurrence type III	1 (20)	
Recurrence type IV	1 (20)	
Primary type II		2 (4)
Recurrence type I	0 (0)	
Recurrence type II	1 (50)	
Recurrence type III	1 (50)	
Recurrence type IV	0 (0)	
Primary type III		15 (28)
Recurrence type I	1 (7)	
Recurrence type II	3 (20)	
Recurrence type III	11 (73)	
Recurrence type IV	0 (0)	
Primary type IV		23 (42)
Recurrence type I	0 (0)	
Recurrence type II	9 (39)	
Recurrence type III	9 (39)	
Recurrence type IV	3 (13)	
Crural disruption	2 (9)	
Primary no HH		2 (4)
Recurrence type I	0 (0)	
Recurrence type II	2 (100)	
Recurrence type III	0 (0)	
Recurrence type IV	0 (0)	
Unkown primary HH		7 (13)
Recurrence type I	0 (0)	
Recurrence type II	1 (14)	
Recurrence type III	6 (86)	
Recurrence type IV	0 (0)

**Fig. 2:**
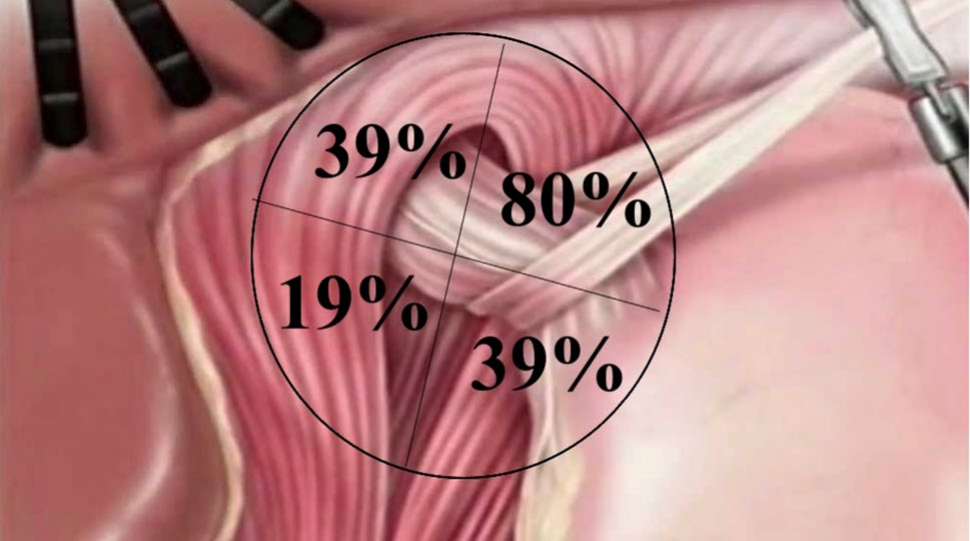
location of recurrent hiatal herniation, divided in quadrants towards the esophagus. Percentages exceed 100% as recurrent hiatal hernia was mostly located in more than one quadrant

Intraoperative identification of the crural defects identified involvement of the right-anterior quadrant in 21 cases (39%), the left-anterior quadrant in 43 cases (80%), the left-posterior quadrant in 21 cases (39%) and the right-posterior quadrant in 10 cases (19%), see Fig. 2. Involvement of more than one quadrant was noted in 37 patients (69%).

## Discussion

In this study, we showed that the left anterior quadrant of the hiatus is affected in 80% of patients who underwent revision surgery for a recurrent hiatal hernia. This finding is consistent with earlier published reports on location of crural defects in recurrent hiatal hernias. A study from Suppiah et al. showed that the anterior part of the hiatus is increasingly involved in recurrent hiatal hernia over time and a recent report from Saad et al. confirmed our finding that the anterolateral quadrant is by far the most affected quadrant in recurrent hiatal hernias [14, 15]. These studies used the operation notes to determine the location of recurrence, where we added the intra-operative videoclip assessment of revisional procedures to determine the type and location of recurrent hiatal hernias. This method allowed a more detailed and repeated assessment of the hiatus. Along with confirmation of findings using operation notes and postoperative imaging, this created reliable and reproducible results. A more recent study by Linnaus et al. reported similar methods by also using video analysis to score the location of HH recurrence [18]. Identical to our study, anatomical quadrants were used, however, involvement of two adjacent quadrants was further classified, e.g., involvement of the left- and right-anterior quadrants classifies as an anterior recurrence. In this study, anterior recurrences were found to be most common (67%) followed by circumferential recurrences in which three or more quadrants were involved (29%).

The finding that the left anterior quadrant is involved in 80% in recurrent hiatal hernia is an important step in developing new effective treatment strategies to reduce the recurrence rate. Recurrence after large hiatal hernia repair is relatively common with numbers increasing up to 57% on long term follow-up [19]. Most recurrent HH are small and may be asymptomatic or will result minor symptoms. A significant part of recurrent HH will require medication, mainly proton-pump inhibitors. When symptom are insufficiently controlled by medication, surgical revision. Several treatment strategies to reduce recurrence rates have been deployed in the past. A commonly used strategy is to reinforce the crural repair with a (non-) absorbable onlay mesh. This strategy does not pursue tension-free repair, but tends to create extra tensile strength at the hiatus. A recent systematic review and meta-analysis of seven randomized controlled trials investigated the effect of mesh reinforcement of crural repair on short- and long-term recurrence rates [20]. In almost all included studies, the mesh was positioned and fixated at the posterior hiatus, leaving the anterior part untouched (see Table 4). Our results suggest that this strategy is not effective to prevent recurrence, as the majority of these recurrences is located anteriorly. It is therefore not surprising that no significant difference was found in the recurrence rate between primary crural repair and onlay mesh reinforcement at long-term follow up [20].

**Table 4:** Overview from the studies analyzed by Petric et al. [20] including group number, longest duration of follow-up, mesh type and mesh position

Study	Numbers mesh/ suture repair	Longest follow up (months)	Mesh type	Mesh position
Frantzides et al. [5]	36 / 36	6–72	Non-absorbable (PTFE)	Semicircular (keyhole)
Granderath et al. [25]	50 / 50	3–12	Non-absorbable (polypropylene)	Posterior onlay
Oelschlager et al. [26]	51 / 57	9–59	Absorbable (Surgisis)	U-shape posterior and lateral onlay
Ilyashenko et al. [27]	50 / 48	36–60	Non-absorbable (polyester)	Posterior onlay
Oor et al. [8]	36 / 36	3–12	Non-absorbable (polypropylene)	Posterior onlay
Analatos et al. [28]	82 / 77	36	Non-absorbable (PTFE)	Posterior onlay
Watson et al. [9]	83 / 43	36–60	Both non-absorbable (polypropylene) and absorbable (Surgisis)	Posterior onlay

Even though recurrences are mostly located anteriorly, the posterior segment is also frequently affected. Studies from Saad et al. and Suppiah et al. showed that the posterior hiatus is typically involved in early types of recurrent hiatal hernias, once the posterior crural repair fails [14, 15]. Anterior recurrences develop gradually over time, thus a recurrence is more a symptom of disease progression rather than a treatment failure of the hiatal repair (with or without mesh reinforcement). Continuing stress on the central tendon of the diaphragm and ageing will lead to a moment where the tensile strength of the anterior part of the hiatus will be insufficient [14]. These ideas are supported by the results from Linnaus et al., who reported that anterior recurrences were more commonly associated with hiatal dilation as mechanism of recurrence compared to all other recurrence locations. Furthermore, reoperations for patients with posterior hiatal repair disruption occurred much sooner following prior repair when compared to patients with hiatal dilation as mechanism of recurrence [18]. The ideal strategy to prevent recurrence therefore seems to be a circular reinforcement of the hiatus reinforcing all four quadrants. This may reduce both anterior and posterior recurrences.

An important drawback of non-absorbable, synthetic mesh at the hiatus is the risk of fibrosis and shrinkage of the mesh causing esophageal stenosis and intraluminal erosion [21]. Although these mesh-related complications are relatively uncommon, the consequences are devastating. Most patients require major surgery such as esophagectomy or gastrectomy [22]. Location of mesh erosion is not specifically reported in literature, but in our experience, when mesh erosion occurs, anterior, non-absorbable mesh reinforcement was often used at the primary procedure. This may be explained by the ventral angle of the intraabdominal portion of the esophagus and movement with each breath and swallow of the diaphragm in relation to the distal esophagus. It may, therefore, be unsafe to use non-absorbable mesh in a circular fashion around the esophagus. Bio-absorbable mesh seems a safer option. It degrades over time and leaves behind a scaffolding for surrounding tissue to grow into. Therefore, it adds additional strength to the crural repair. A recent meta-analysis investigating the effectiveness of the bio-absorbable mesh showed a significant lower recurrence rate at the mid-term when compared to simple suture cruroplasty. The bio-absorbable mesh however was placed posterior to the esophagus in a U-shape instead of circular around the esophagus. Nonetheless, studies were reported to be very heterogenous, leading to the authors pleading for further studies investigating the long-term outcomes while accounting for various factors such as hernia classification, surgical technique and post-operative outcomes [23]. Further research is needed before the biological mesh is ruled a success since too little evidence is available at this moment. We have initiated a randomized controlled trial investigating the effectiveness of a circular, bio-absorbable mesh in our center, taking into account the factors described above.

Although the biological mesh shows promising results, it is important to acknowledge other mesh options available for anterior hiatal reinforcement. In a study by Akmaz et al. the effectiveness of vertical mesh strips (VMS) was investigated. This small prosthetic strip is placed vertically over both hiatal pillars and was associated with lower recurrence rates in combination with additional anterior cruroplasty. Additionally, fewer mesh related complications were reported compared to other prosthetic meshes due to the smaller surface area [24]. It is safe to say further research is needed on both prosthetic and bio-absorbable reinforcement options to determine the optimal technique.

A limitation to our study concerns the inclusion of only patients who underwent a revisional procedure for a recurrent HH. As mentioned before, not all recurrent HH have symptoms that require surgical revision. A proportion of the cases that do produce symptoms can be dealt with through medication, which narrows our population down to recurrent HH cases with significant symptom burden. This is furthermore reflected by the smaller population size. Therefore, our study results may not be applicable to all recurrent HH. However, since asymptomatic recurrent HH are of lower clinical importance and our findings are consistent with other reports, we believe this did not significantly impact the validity of our results.

## Conclusion

In our study, we investigated the anatomic location of the recurrent hiatal hernia and tried to understand why the recurrence rate after surgical repair remains high, even after mesh augmentation. We conclude that in our cohort, the left-anterior quadrant of the esophageal hiatus is mostly involved in hiatal hernia recurrences. Most RCTs investigating the effectiveness of mesh augmentation leave the anterior part of the hiatus untouched. After comparing our results to other studies investigating the location of HH recurrences, we plead for the reinforcement of both the posterior and anterior hiatus during HH repair. We hypothesize that a circular mesh reinforcement supporting all four quadrants of the esophageal hiatus may reduce the high-recurrence rate after primary suture repair for HH. We have initiated a randomized controlled trial using a circular, biodegradable mesh to test our hypothesis.
